# Ten cold clubfeet

**DOI:** 10.1080/17453674.2018.1493046

**Published:** 2018-07-09

**Authors:** Robert B Giesberts, Edsko E G Hekman, Gijsbertus J Verkerke, Patrick G M Maathuis

**Affiliations:** 1 University of Twente, Department of Biomechanical Engineering, Enschede;; 2 University of Groningen, University Medical Center Groningen, Department of Rehabilitation Medicine, Groningen;; 3 University of Groningen, University Medical Center Groningen, Department of Orthopaedic Surgery, Groningen, the Netherlands

## Abstract

Background and purpose — Idiopathic clubfeet are commonly treated with serial manipulation and casting, known as the Ponseti method. The use of Plaster of Paris as casting material causes both exothermic and endothermic reactions. The resulting temperature changes can create discomfort for patients.

Patients and methods — In 10 patients, we used a digital thermometer with a data logger to measure below-cast temperatures to create a thermal profile of the treatment process.

Results — After the anticipated temperature peak, a surprisingly large dip was observed (*T_min_* = 26 °C) that lasted 12 hours.

Interpretation — Evaporation of excess water from a cast might be a cause for discomfort for clubfoot patients and subsequently, their caregivers.

The common treatment of idiopathic clubfoot (talipes equinovarus) consists of serial manipulation and casting, known as the Ponseti method (Ponseti 2008). The treatment is started in the first weeks after birth and includes on average 5 cast changes, often followed by a percutaneous Achilles tenotomy. The deformity is successfully corrected in over 90% of all cases (Morcuende et al. 2004). An abduction orthosis is worn for several years to prevent relapse (Dobbs et al. 2004).

The Ponseti method dictates toe-to-groin casts with the knee flexed to prevent slipping of the cast (Ponseti 2008, Maripuri et al. 2013). Plaster of Paris (PoP) is the accepted casting material, because it is inexpensive, easily obtained, and it can be easily molded (Aydin et al. 2015, Pittner et al. 2008). The use of PoP as casting material involves both an exothermic (hot) setting reaction and an endothermic (cold) drying process.

Bandage rolls impregnated with PoP are soaked in lukewarm water before being wrapped around the foot. The PoP has an exothermic chemical reaction with water. Many studies have focused on the extent of the generated heat and the effects of the associated temperature peak, which appears minutes after application (Shuler and Bates 2013, Burghardt et al. 2014). Thermal injuries can occur if the temperature exceeds 40 °C for longer periods of time, but can be prevented by optimizing the number of layers used, cast type and brand, dipping water temperature, and cast padding thickness (Halanski et al. 2007, Hutchinson and Hutchinson 2008, Shuler and Bates 2013). Only a few layers of cast tape are needed to keep the tiny clubfeet in position, therefore thermal injuries are uncommon in the Ponseti method.

Evaporation of the excess water in the cast is an endothermic process, meaning that it extracts energy from its surroundings, thereby cooling it. The required energy for the evaporation of the excess water—the latent heat—is drawn in part from the patient. The effect of this endothermic reaction in the treatment of clubfoot has not been studied before. However, anecdotal information from parents suggests that some children struggle to keep warm with the wet and cold casts (Bridgens and Kiely 2010, van Doorn 2016).

We assessed the below-cast temperature profile in the treatment of clubfoot with the Ponseti method, in terms of both temperature drop and the extent of this drop.

## Patients and methods

### Children

10 children with idiopathic clubfoot, who were younger than 3 months old, and who had had no form of prior treatment were selected for this study (Table 1). From the children who presented with bilateral clubfoot only 1 foot was included for measurements.

**Table 1.** Child characteristics. Unless stated otherwise values are presented as mean (SD)

Characteristic	Value
Children	10
Age (range)	7 (2–30) days
Boys/girls	9/1
Bilateral	6
Pre-treatment Pirani score	4.3 (0.7)
Pre-treatment Diméglio score	14.0 (1.6)

### Sensors

A 1-wire DS1825 digital thermometer (Maxim Integrated, San Jose, CA, USA) was used to measure the temperature under the cast. The accuracy of the sensor is ±0.5 °C (Maxim Integrated 2005).

### Protocol

**Figure 1.**
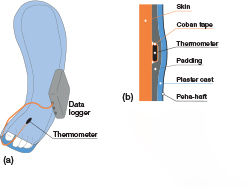
Thermometer location. (a) Location of the thermometer on the foot, below the cast. The plaster is represented in blue. The casing for the data logging system was attached to the cast with a layer of Peha-haft bandage. (b) Schematic representation of the layers between the skin and the thermometer.

After manipulation of the foot, the orthopedist placed the thermometer on the plantar aspect of the foot. A strip of Coban tape (3M, Neuss, Germany) was used between the skin and the thermometer and wires (Figure 1). The thermometer was connected to an electronic data logging system with a small battery and SD card, enclosed in 3D-printed casing. After placing the sensor, the foot was treated as usual following the Ponseti method.

The cast technician cut 1 roll of Gypsona (BSN Medical GmbH, Hamburg, Germany) in half, dipped it in a sink filled with lukewarm (20–25 °C) water and applied it to the clubfoot while the orthopedist maintained the position of the foot. Care was taken not to use more than 3 layers of cast tape. Wires were routed distally and, as soon as the plaster had set, the casing was attached to the cast laterally with a single layer of Peha-haft bandage (Hartmann, Heidenheim, Germany) (Figure 1a). Parents were instructed to elevate the legs of the child with a rolled-up towel, as protocol directs.

At the next scheduled weekly meeting the cast and thermometer were removed. While the parents bathed their child, the sensors were left in a stable position for at least 15 minutes for calibration after which the data were copied to a computer and the battery replaced. The foot was carefully inspected for any signs of skin damage and the protocol was repeated until the final cast before tenotomy.

### Water content

The same measurement protocol was used to cast 1 rubber clubfoot model (MD Orthopaedics, Inc., Wayland, IA, USA). The weight of all components was documented and the total weight of the rubber foot plus cast was monitored for several days. This provided an estimation of the excess water content in the plaster cast and the time it takes to evaporate.

The latent heat of evaporation of water is described as *Q = m·*Δ*Hev*, with *m* the measured mass of the excess water and Δ*Hev*(25 °C) = 2,442 J/g (Rajput 2010).

### Data collection and data processing

The system was programmed to store time and temperature data continuously (0.16 Hz) for the first 4 hours, after which the system entered a low power state (85 µA) to save battery power. 4 times per hour it woke from this state to collect data for 10 seconds. Each 10 s period resulted in 2 temperature measurements over which the average was taken. The system featured a button to wake up after the cast was removed.

Data was processed using Matlab version R2016b. Potential sensor errors were filtered using the median filter *medfilt1* (Pratt 2007). The start of the measurement (*t_0_*) was defined as the moment the plaster cast touched the foot. The height of the temperature peak (*T_max_*) was calculated as the maximum value over the first 3 hours, the final equilibrium temperature (*T_end_*) as the mean value over the final 3 days of measurement, and the lowest temperature (*T_min_*) as the minimum value after *T_max_*.

The beginning of the temperature dip was defined as the moment the temperature reached a value smaller than 1 standard deviation of *T_end_*. The moment the temperature reached a value within 1 standard deviation of *T_end_* was defined as the end of the temperature dip.

The 3 sets of temperature data (*T_max_, T_min_* and *T_end_*) were tested for normality using the Shapiro-Wilk test. The Student t-tests were used to test the difference between temperatures for statistical significance.

## Ethics, funding, and potential conflicts of interest

The medical ethical evaluation committee of the UMCG reviewed the study in accordance with the declaration of Helsinki, and declared that it did not meet the criteria as stated by the Medical Research Involving Human Subjects Act (WMO), and therefore did not require their approval (document number M16.196266). After obtaining informed written consent from both parents, the children were included in the study. Financial support was provided by Stichting voor de Technische Wetenschappen [grant P12-03]. No competing interests declared.

## Results

Complications in the form of pressure marks were encountered in 4 cases. In 2 of those cases further measurements were canceled but the data obtained were included for analysis.

Technical malfunctioning occurred in 5 measurements (disconnected sensors, disconnected thermometer, faulty measurement protocol, empty battery). 29 successful measurements were performed on 10 clubfeet, giving more than 3,000 hours of data.

### Clubfoot model—water content

The measurement with a clubfoot model showed that approximately 35 g of water was used for one roll of PoP bandage of 75 g. Over the course of 2 days the water content in the cast decreased to reach an equilibrium of 14 g. This means that 14 g of the original water content attached to the PoP to form hard gypsum and the remaining 21 g of excess water evaporated. This ratio fits well within the molecular ratio of the chemical reaction, which also predicts the generation of 740 kJ of heat (Table 2). The required energy for evaporation of 21 g of excess water is 51 kJ.

**Table 2.** Molecular ratios

	CaSO_4_ (H_2_O)_1/2_ + 1½H_2_O → CaSO_4_(H_2_O)_2_ + H_2_O + heat				
Molecular mass (mol^-1^)	141.15 g	1½×18.015 g	172.17 g	18.015 g	1,433 kJ
Mass (mol)	0.52	0.78	0.52	Excess ^a^	0.52
Mass (g)	75	14	89	21	740 kJ

a The 21 g of excess water measured fits the prediction based on the molecular ratios of the exothermic setting reaction.

### Clubfoot measurements

**Figure 2.**
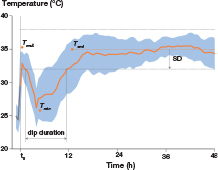
Results from the temperature measurements. The orange line indicates the average temperature over all measurements, the blue area the standard deviation (SD).

All temperature measurements showed a clear temperature peak after the application of the plaster cast (*T_max_*). None of the below-cast temperatures ever exceeded 40 °C and no thermal injuries were observed (Figure 2 and Table 3).

**Table 3.** Temperature data

	Time (hours) median (IQR)	Temperature (°C) mean (SD) [95%CI]	Normality ^a^ p-value
*T_max_*	0.15 (0.1–0.8)	35.3 (1.6) [34.7–35.8]	0.5
*T_min_*	4.9 (4.0–7.3)	26.1 (3.0) [25.0–27.2]	0.2
*T_end_*	11.0 (9.6–15.8)	34.9 (1.4) [34.4–35.5]	0.09

a Temperature data was tested for normality using Shapiro–Wilk tests.

**Figure 3.**
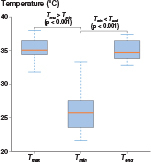
Difference in the measured temperature. *T_max_* represents the temperature peak shortly after casting, *T_min_* the lowest temperature reached after the initial peak, and *T_end_* the equilibrium temperature.

After the peak, a decrease to a lower temperature was observed (p < 0.001) (Figure 3). On average, this temperature dip lasted 12 (range 3.2–30) hours followed by a gradual increase to a stable final temperature (*T_end_*).

All 3 sets of temperature data were normally distributed (Table 3). There was no statistical difference between the temperature peak and the equilibrium temperature (p = 0.3).

## Discussion

This is the first study to present long-term temperature measurements underneath the cast in the treatment of clubfeet. A surprisingly large temperature dip was observed caused by the evaporation of excess water. In several cases temperatures as low as 22 °C were observed and they stayed low for more than 12 hours.

### Evaluation of the results

**Figure 4.**
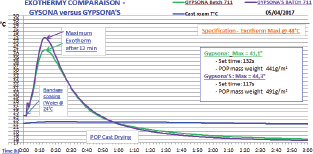
Gypsona temperature profile. Measurements from BSN Medical show the steep increase and subsequent gradual decline of the temperature after application. Copied with permission from BSN Medical.

Our measurements showed a temperature peak after 9 minutes (Table 3). BSN Medical’s own measurements with Gypsona show a peak after 12 minutes (Figure 4). Burghardt et al. (2014) found a peak temperature after 9 minutes when using 16 layers of a different brand of plaster.

Immediately after reaching the peak, the cast started to cool. This is also visible in BSN Medical’s measurement in which the temperature drops below the ambient temperature after approximately 1 hour (Figure 4). Depending on environmental influences it can take 2 to 3 days for the cast to dry completely (Hayter 2015). Indeed, in our water content measurement the cast reached a stable weight after 2 days. However, on the clubfeet it took an average of 11 hours before the temperature reached normal levels again. This suggests that the plaster dried much quicker when placed on the child’s foot and that some of the child’s energy was used to evaporate the water of the plaster cast.

### Casting-induced hypothermia

In adults, 1 case study exists which describes observed hypothermia after the application of a plaster jacket for immobilization of the cervical spine (Vale 1969). Caregivers of children with a clubfoot have mentioned that their children can be a bit prickly in the first 24 h after a new cast is applied (Bridgens and Kiely 2010, van Doorn 2016). The observed cold period might be one of the causes for this behavior.

It would take 51 kJ to evaporate the excess water of 1 cast. Part of this energy will be extracted from the environment, but a substantial part of it must be produced by the child. However, the thermoregulatory system of newborns is not yet fully developed, and they experience great difficulties when facing a cold environmental challenge (Pierro et al. 2012). Moreover, newborns have a relatively large surface area, so much heat is lost in relation to their heat-producing volume.

A newborn’s total energy expenditure in the first month after birth is 1.0–1.3 MJ/day (FAO 2001, Olgaher and Forsum 2003). Up to 8% of this energy—approximately 100 kJ/day—is used for thermogenesis and thermoregulation (Pierro et al. 2012). Relatively, the required 51 kJ for the evaporation of excess water is quite substantial and a mild form of casting-induced hypothermia in the treatment of clubfoot seems plausible, especially with bilateral clubfeet in vulnerable patients such as prematurely born children. In those cases, postponing treatment at least until after the first month might be beneficial (Alves et al. 2009, Awang et al. 2014, Íltar et al. 2010).

Given that newborns are naturally well monitored by their parents, that hypothermia in newborns is easily detected, and that no such case studies exist for clubfoot, it is unlikely that casting induces severe or moderate hypothermia. Still, having cold feet for half a day undoubtedly is uncomfortable and plausibly affects the newborn’s mood.

## Limitations

We acknowledge limitations of our study. First, we did not measure whether the wet and cold casts affect the child’s core temperature. If they do, a more prominent effect would be expected in bilateral cases. However, the number of children in our study does not allow for any meaningful test for subgroup differences, and no trend could be identified.

Second, the sensors were placed on the skin on the plantar aspect of the foot. So the measurements reflect only the temperature of that part of the foot and not the core temperature of the patient. Hypothermia was neither measured nor diagnosed based on clinical symptoms. Future research should include measuring the core temperature during the first 24 hours after casting to study the possibility of casting-induced hypothermia.

## Clinical implications

So far only anecdotal information exists regarding cold clubfeet (Bridgens and Kiely 2010, van Doorn 2016). In some cases, especially with bilateral clubfeet in vulnerable children, it might be better to postpone treatment to prevent any harmful effects of the wet and cold casts. This is an extra argument to defer treatment in premature babies for several weeks, as stated by the First European consensus meeting on Ponseti clubfoot treatment’s agreement (Böhm and Sinclair 2013).

Preventing a clubfoot from getting cold would be possible if less excess water needed to be evaporated. However, squeezing the water out of the plaster to use less water results in hotter plaster and increased risk of burns: the excess water is needed to release the heat from the exothermic reaction (Kaplan 1981).

Using synthetic cast tape as an alternative casting material could be a viable option to decrease the discomfort of cold feet. The chemical reaction and the structure of the material both cause the drying process to be shorter than when using PoP.

In summary, after an anticipated temperature peak, a surprisingly large temperature dip was observed. More research is needed to assess whether the Ponseti method affects the child’s core temperature.

The authors would like to thank the parents of all participants for their trust and patience, and the medical staff of the Gipskamer for all their help and advice. Special thanks are offered to Maaike Vos, Els Huizenga, and Rohan Choudhari for performing the measurements.

RG conceived and designed the experiments, performed the experiments, analyzed the data, and wrote the paper. EH and GV critically edited and reviewed the test protocol, data interpretation, and the manuscript. PM performed the experiments, critically edited and reviewed the test protocol, data interpretation, and the manuscript.

*Acta* thanks Naomi Davis and Henrik Wallander for help with peer review of this study.
